# Comparative performance of SARS-CoV-2 real-time PCR diagnostic assays on samples from Lagos, Nigeria

**DOI:** 10.1371/journal.pone.0246637

**Published:** 2021-02-04

**Authors:** Chika Kingsley Onwuamah, Azuka Patrick Okwuraiwe, Olumuyiwa B. Salu, Joseph O. Shaibu, Nnaemeka Ndodo, Samuel O. Amoo, Leona C. Okoli, Fehintola A. Ige, Rahaman A. Ahmed, Munir Akinwale Bankole, Judith O. Sokei, Bamidele Paul Mutiu, James Ayorinde, Babatunde Akeem Saka, Celestina Obiekea, Nwando Mba, Richard A. Adegbola, Sunday Omilabu, Chikwe Ihekweazu, Babatunde Lawal Salako, Rosemary Audu

**Affiliations:** 1 Microbiology Department, Centre for Human Virology and Genomics, Nigerian Institute of Medical Research, Yaba, Lagos State, Nigeria; 2 Department of Medical Microbiology and Parasitology, Centre for Human & Zoonotic Virology, College of Medicine, University of Lagos, Idi-Araba, Lagos State, Nigeria; 3 National Reference Laboratory, Nigeria Centre for Disease Control, Abuja, Nigeria; 4 Lagos State Biosecurity and Biobank Laboratory, Mainland Hospital, Lagos State Ministry of Health, Lagos, Lagos State, Nigeria; 5 Department of Biochemistry and Nutrition, Nigerian Institute of Medical Research, Yaba, Lagos State, Nigeria; 6 Global Emerging Pathogens Treatment Consortium (GET), Yaba, Lagos State, Nigeria; 7 Director-General’s Office, Nigeria Centre for Disease Control, Abuja, Nigeria; 8 Director-General’s Office, Nigerian Institute of Medical Research, Yaba, Lagos State, Nigeria; Alagappa University, INDIA

## Abstract

A key element in containing the spread of the SARS-CoV-2 infection is quality diagnostics which is affected by several factors. We now report the comparative performance of five real-time diagnostic assays. Nasopharyngeal swab samples were obtained from persons seeking a diagnosis for SARS-CoV-2 infection in Lagos, Nigeria. The comparison was performed on the same negative, low, and high-positive sample set, with viral RNA extracted using the Qiagen Viral RNA Kit. All five assays are one-step reverse transcriptase real-time PCR assays. Testing was done according to each assay’s manufacturer instructions for use using real-time PCR platforms. 63 samples were tested using the five qPCR assays, comprising of 15 negative samples, 15 positive samples (Ct = 16–30; one Ct = 35), and 33 samples with Tib MolBiol E-gene Ct value ranging from 36–41. All assays detected all high positive samples correctly. Three assays correctly identified all negative samples while two assays each failed to correctly identify one different negative sample. The consistent detection of positive samples at different Ct/Cq values gives an indication of when to repeat testing and/or establish more stringent in-house cut-off value. The varied performance of different diagnostic assays, mostly with emergency use approvals, for a novel virus is expected. Comparative assays’ performance reported may guide laboratories to determine both their repeat testing Ct/Cq range and/or cut-off value.

## Introduction

The novel coronavirus, SARS-CoV-2 became a pandemic in the year 2020, and many nations shut down to contain its spread. A key element in containing its spread is to identify infected people, trace their contacts, and have them quarantined in treatment and isolation centers, or through self-isolation, especially if they are asymptomatic.

Daily the world is gathering more information about SARS-CoV-2, and many diagnostic assays have been developed to identify those infected. These assays have varied performance in correctly identifying people infected and those not infected. Current knowledge shows the variability between released SARS-CoV-2 genomes is small, being >99% related, which would be an aberration as RNA-viruses are often prone to mutations [1,2]. Furthermore, current data suggests the virus evolves differently in diverse populations [3]. Several viral mutation hotspots have been identified, including those able to affect available diagnostics [1,2,4]. Two mutation hotspots have been identified within the coding regions [1], involving the ORF1ab mostly targeted by currently available diagnostics [1,2,4]. If mutation(s) causes misdiagnosis, it can lead to missed infections that could fuel the spread of the virus as diagnostics perform below acceptable levels [2,4].

Most real-time PCR assays for detecting new infections use a variety of sample types, mostly nasal and oropharyngeal swabs. Across the world, various diagnostic assays have been developed, and over 200 different assays were submitted to the Foundation for Innovative New Diagnostics (FIND) for independent evaluation and comparison with the Tib MolBiol assays as the reference [5]. Preliminary evaluation results have been released on their website and a modified version is shown in Table 1 [5]. Five different real-time PCR diagnostic assays for SARS-CoV-2 were used in this study: BGI (BGI Health (HK) Co. Ltd, China), Da An Gene (DAAN Gene Co. Ltd, China), Genesig (Primerdesign Ltd, United Kingdom), Liferiver (Liferiver Bio-Tech (United States) Corp.) and Tib MolBiol (TIB MOLBIOL, GmbH, Germany) real-time PCR diagnostic assays. All these assays are one-step reverse transcriptase PCR (RT-PCR) assays. FIND has completed and published online the preliminary evaluation results for four of the five diagnostic assays (Table 1), namely the BGI, Da An Gene, Primerdesign Genesig and Tib MolBiol real-time PCR diagnostic assays [5].

**Table 1:** FIND evaluation results for first set of COVID-19 diagnostic assays.

Company	Gene target	Verified LOD (copies/reaction)	Average Ct (lowest dilution 10/10)	Clinical sensitivity (50 positives)	Clinical specificity* (100 negatives)	Product No.	PCR platform Used for evaluation	Supplier recommended Ct cut-off
**BGI Health (HK) Co. Ltd**	ORF1	1–10	32^.^43	100% (95%CI: 93, 100)	99% (95%CI: 95, 100)	MFG030010	Roche LightCycler 480	≤38
**DAAN Gene Co. Ltd**	ORF1	1–10	38^.^76	100% (95%CI: 93, 100)	96% (95%CI: 90, 98)	DA0930-DA0932	Roche LightCycler 480	≤ 40
	N	1–10	36^.^97	100% (95%CI: 93, 100)	98% (95%CI: 93, 99)			
**Primerdesign Ltd**	RdRP	1–10	36^.^7	100% (95%CI: 93, 100)	100% (95%CI: 96, 100)	JN-02780-009	LightCycler 480	Any signal regarded as positive
**Tib Molbiol (Reference assay)**	E	1–10	33^.^34	100% (95%CI: 93, 100)	100% (95%CI: 96, 100)	53-0776-96 6754155001	Roche LightCycler 480	Cut-off as 2–4 cycles above observed Cp value for 10 copies

*Further investigation needed to determine if apparent false positives are truly false positives or whether they are a due to a false negative reference standard result. (https://www.finddx.org/covid-19/sarscov2-eval-molecular/molecular-eval-results/).

The BGI assay uses ten μl of sample RNA to detects the SARS-CoV-2 ORF1 region, with an internal control detecting a human housekeeping gene [6]. If detected, this internal control helps to verify that sampling was done correctly and precluded a negative sample from being false negative or inhibited. The assay run validation includes ensuring curves are S-shaped, no Ct/Cq values for the blank control and both targets detected for the positive control with Ct/Cq ≤ 32 [6]. Similarly, each sample tested must have the internal control detected at Ct/Cq ≤ 32 to be accepted as a valid test. The limit of detection is 100 copies / mL with inter- and intra-assay precision less than 5% [6].

The Da An Gene assay detects two SARS-CoV-2 targets (Table 1) and an internal standard gene using five μl of sample RNA [7]. The assay run validation includes no SARS-CoV-2 Ct/Cq values for the blank control but a Ct value for the internal control [7]. For positive control, both SARS-CoV-2 targets must be detected at Ct/Cq ≤ 32 [7]. However, for the positive control and samples, the detection of the internal standard gene is not compulsory due to competition [7]. Detection of the internal standard gene is only mandatory for negative samples with no Ct for both SARS-CoV-2 targets [7]. If only one target for SARS-CoV-2 is detected, the test is repeated, and if consistent, the sample is deemed positive, else deemed negative [7]. The declared analytical sensitivity is 500 copies / mL and precision less than 5% [7].

The Genesig assay detects the SARS-CoV-2 RdRP gene (Table 1) using eight μl of sample RNA. It also detects an RNA extraction control that validates extraction and PCR processes. The positive control should have a Ct between 14–22 in the FAM channel, while the negative extraction control (NEC) should have a CT <30 for the assay to be considered valid [8]. The CT of the NEC is critical in deciding infection status. The Genesig assay detects 0^.^58 copies /μL with ≥ 95% confidence [8]. Its intra-assay precision ranged from 0^.^72–0^.^91% [8]. Inter-assay precision between qPCR instruments, different operators and different days ranged from 6^.^98–8^.^53% [8]. Using 50 positive and 50 negative samples, Genesig had 98% and 100% agreement, respectively [8]. The utility of the Genesig assay is that being lyophilised, it can be shipped at ambient temperature but should be stored at -20°C on arrival [8].

The Liferiver real-time multiplex RT-PCR assay uses five μl of sample RNA to detect three SARS-CoV-2 genes, namely the ORF1ab, the N-gene and the E-gene [9]. It further detects an internal control. The primers and probes for the assay were designed to detect six SARS-CoV-2 strains [9]. Its analytical sensitivity is 1000 copies/mL [9]. For each run to be valid, the negative control should have a CT value between 25–40 for the internal control, while the positive control should have a CT value ≤35 for all the three SARS-CoV-2 genes [9]. Detection of the internal control is not a requirement for the positive control or positive samples but a requirement for the negative control or negative samples [9]. Positive samples must have the ORF1ab detected, along with the N-gene and/or E-gene [9]. Detection of any other combination of genes, including both N-gene and E-gene without ORF1ab gene, gives an inconclusive result. Cut-off CT for a positive sample is 41 [9].

The Tib MolBiol method consists of two different assays detecting first the E-gene and subsequently the RdRP-gene for those positive on the first assay. The assay can use five–ten μl of sample RNA. The E-gene detects a 76bp fragment of SARS and SARS-CoV-2 E-gene [10]. The manufacturer stated the E-gene assay detects a minimum of 10 copies [10]. The method provides a 70bp Equine Arteritis Virus (EAV) genome to spike in the samples before extraction, and this is subsequently detected as the extraction internal control. Detection of the EAV internal control is compulsory for the negative control and negative samples while it is not relevant for the positive control and positive samples [10,11]. Samples positive by the E-gene assays are subsequently confirmed in a test detecting 100bp fragment of the RdRP gene-specific to SAR-CoV-2 [11]. The manufacturer stated the RdRP-gene assay detects ten copies and recommends that cut-off be set at 1–2 cycles higher than observed crossing point for ten copies.

As an ISO 15189:2012 accredited facility and a World Health Organization Prequalification testing facility, we are concerned with the establishment and maintenance of quality standards in laboratory workflows. We now report the findings from testing the same sample panel, obtained from persons presenting for diagnosis of SARS-CoV-2 infection in Lagos, Nigeria, on five different diagnostic assays with recommendations to assure high quality output.

## Materials and methods

This retrospective study compared five real-time one-step reverse transcriptase PCR assays for detecting SARS-CoV-2. All assays were performed according to the manufacturers’ instructions.

### Study design and site

The Nigerian Institute of Medical Research (NIMR) established a drive-through test centre for SARS-CoV-2 in March 2020, where suspected persons visited for testing. This assay comparison was performed at its ISO 15189:2012 accredited Centre for Human Virology and Genomics between April–May 2020. This Centre is also a World Health Organization Prequalification evaluating laboratory. In a collaboration with China CDC, real-time PCR test kits made by BGI were received. After, the integration of NIMR into the Nigeria Centre for Disease Control (NCDC) testing network in March, two other real-time PCR test kits namely the Tib MolBiol MDx and Da An Gene assay were further received. Subsequently, a donation of the Genesig and Liferiver real-time PCR diagnostic assays was received. These five assays were used to test samples to determine their comparative performance. Results for clinical diagnosis used for the management of the clients were given out based on the kit in routine use at the time and its manufacturer’s instructions for use.

### Study population

Samples received at the laboratory for SARS-CoV-2 diagnosis were utilised in this analysis. These samples were from people living in Lagos, Nigeria, who were seeking a diagnosis for SARS-CoV-2 during the peak of the pandemic, from the last week of March 2020 to the second week of April 2020. People coming to the Drive-through/Walk-in testing Centre for diagnosis were mostly asymptomatic, though very few symptomatic elderly persons were attended to. The population varied with the pandemic timeline, first being mostly travellers and their contacts, then expanding to health workers and people with fever or cough as community transmission began. As samples were deidentified prior to use, we do not have access to specific patient data. These samples included those negative and positive for SARS-CoV-2, especially the presumed low positives samples often on the borderlines of the cut-off CT values of the various assays. The negative and positive samples were determined using the Tib MolBiol assay, while the borderline samples were beyond the Tib MolBiol E-gene assay cut-off but determined as positive on one or more other assays.

### Laboratory methods

The Centre had the unique privilege of having five different diagnostic methods for SARS-CoV-2. It utilised these assays to determine their comparative performance on samples taken from people in Nigeria who were being assessed for possible infection with the virus.

The QuantStudio 3 (Thermofisher), the CFX Connect (Bio-Rad) and the CFX96 Deep Well (Bio-Rad) were the real-time PCR platforms utilised for the laboratory analysis. The study was started with the QuantStudio 3 and expanded to utilising the CFX platforms as the pressure for testing increased.

For quality control and quality assurance, negative/blank and positive controls for each assay were aliquoted into 0^.^2 mL PCR tubes in volumes suitable for single-use during laboratory analysis to eliminate contamination and degradation of the control materials through frequent freeze-thaw cycles. Similarly, on opening each assay box, where necessary depending on the run size, an aliquot was made and were stored at -20°C. A template specifying the qPCR run conditions for each assay was developed for the three qPCR platforms. Following the manufacturer’s instruction and wherever acceptable, the baseline threshold setting was automated and verified during the review of each run. The automation of threshold setting minimised variation in assay parameters that could affect the Ct/Cq values. The blank/negative and positive controls of each run must meet the assay requirements before a run is accepted as valid.

RNA from the samples used for testing were extracted using the Qiagen Viral RNA Kit (Qiagen Inc, Valencia, CA, USA). According to the manufacturer’s instruction, 140 μL of the viral transport medium containing the nasopharyngeal swabs were used for RNA extraction and eluted in 60 μL of AVE buffer. These were stored at -20°C in between same-day use and at -80°C for long period storage at the laboratory. Test determinations were first performed for the BGI, Da An and Tib MolBiol qPCR assays in April 2020 using freshly extracted and characterised samples. Testing with the Genesig and Liferiver qPCR assays were done in May 2020 using the well-preserved RNAs samples. The real-time PCR analysis were all done according to the manufacturer’s instruction.

### Data analyses

Data analysis to obtain sensitivity, specificity and accuracy were performed using MEDCALC® easy-to-use statistical software free online Diagnostic test version [12].

### Ethical approval and consent to participate

Ethical approval was obtained from the Institutional Review Board of the Nigerian Institute of Medical Research for the Drive-through/Walk-in COVID-19 testing Centre, to cover collecting samples for diagnostic and research purposes (IRB/20/024). In addition, the Nigerian Institute of Medical Research set-up a website for persons seeking diagnosis to forestall the gathering of large crowds. They fill the form online before being invited for sampling and testing, at a specified date and time. All were informed that as a research institution, their de-identified samples may be used for research purposes and they gave their consent willingly. Routinely, samples are coded, and these codes replace any personal identifiers within the testing workflow. We utilised the leftover samples of people who gave consent for the use of their deidentified samples for research.

## Results

A total of 63 samples were tested using the five qPCR assays. Of the fifteen negative samples, the BGI and the Genesig assays determined all as negatives. The Da An Gene assay determined 14/15 samples were negative while one was positive with CT values of 39^.^910 and 37^.^230 on the ORF1ab and N-gene, respectively. However, repeat testing by Da An Gene assay for this one sample gave a negative result. Similarly, the Liferiver assay repeatedly detected another sample as positive for N-gene (Cts = 36^.^707/38^.^406) and E-gene (Cts = 36^.^766/37^.^991). Thus, one sample here had an inconclusive test result from the Liferiver assay.

Of the fifteen high positive samples with Ct values of 23^.^3–31^.^4, all five qPCR assays were concordant on these samples being positive for SARS-CoV-2. The sensitivity, specificity, and accuracy of the five assays were determined using the negative and high positive samples, as shown in Table 2.

**Table 2:** Performance characteristics of the different assays.

Assay	Sensitivity [%] (95% CI)	Specificity [%] (95% CI)	Accuracy [%] (95% CI)
**BGI**	100^.^0 (78^.^2–100^.^0)	100^.^0 (78^.^2–100^.^0)	100^.^0 (88^.^4–100^.^0)
**Da An**	100^.^0 (78^.^2–100^.^0)	93^.^3 (68^.^1–99^.^8)	96^.^7 (82^.^8–99^.^9)
**Genesig**	100^.^0 (78^.^2–100^.^0)	100^.^0 (78^.^2–100^.^0)	100.0 (88.4–100^.^0)
**Liferiver**	100^.^0 (78^.^2–100^.^0)	93.3 (68^.^1–99^.^8)	96^.^7 (82^.^8–99^.^9)
**Tib MolBiol**	100.0 (78^.^2–100^.^0)	100^.^0 (78^.^2–100^.^0)	100^.^0 (88^.^4–100^.^0)

CI = Confidence interval. Our small sample size may not render the best estimate of these parameters for the assays evaluated

For the samples presumed to be low positive samples (n = 33), there were variable results on the different assays (Table 3). The MDx Tib MolBiol E-gene assay has a cut-off of Ct <36^.^0 for positive samples, revised down from the initial 39^.^0 (10). Thus, most samples herein presumed to be low positives would have been declared negative for the SARS-CoV-2.

**Table 3:** Status of samples above Tib MolBiol cut-off on the other assays.

MDx E-gene CT	#	Negative				ORF1 Positive				N-gene Positive		E-gene Positive	Positive on all other assays (%)
		BGI	Da An	Genesig	Liferiver	BGI	Da An	Genesig	Liferiver	Da An	Liferiver	Liferiver	
**36.0–36.9**	6	-	-	0	0	6	6	5*	5*	6	5*	5*	100.0
**37.0–37.9**	3	-	1	1	1	3	2	2	2	2	2	1	66.7
**38.0–38.9**	9	1	1	4	2	8	8	5	7	8	5	6	44.4
**39.0–39.9**	5	1	2	2	0^#^	4	3	3	3	3	3	4	60.0
**40.0–40.9**	7	3	3	5	3	4	4	2	4	3	4	3	14.3
**41.0–41.2**	3	-	0	1	0^#^	3	3	2	2	2	2	2	66.7

*One sample RNA exhausted.

^#^Two/one samples inconclusive (see Table 4).

The four other assays detected all samples with Ct/Cq 36^.^0–36^.^9 on Tib MolBiol’s E-gene assay as positive for the SARS-CoV-2, including full positive status on assays detecting multiple genes. One sample was exhausted and could not be tested by Genesig and Liferiver assays (Table 3).

Across the categories of Ct values, several samples were detected as concordant positive on all the other four assays (Table 3). However, there was only one concordant negative on all other assays, as a second potential concordant negative was inconclusive on the Liferiver assay having detected the E-gene consistently (Table 4). Of these 33 presumably low-positive samples, only one was negative on all four assays (Table 4), while 17/33 (51^.^5%) were all positive on the other four assays. In addition, the two multiplex assays often detected one (Da An Gene assay) or two (Liferiver assay) genes consistently (Table 4). Of the 17 low positive samples detected by the other four assays, 15/17 were positive for both the two targets of Da An Gene assay and for the three targets of the Liferiver assay (Table 4).

**Table 4:** Assays CT values for samples above Tib MolBiol E-gene cut-off.

ID	CT/CQ values							
	MDx	BGI	DaAn		Genesig	Liferiver		
	*E-gene*	*ORF1*	*ORF1ab*	*N-gene*	*ORF1ab*	*ORF1ab*	*N-gene*	*E-gene*
**1**	36^.^0	31^.^1	36^.^6	34^.^2	34^.^6	32^.^8	34^.^0	33^.^3
**2**	36^.^1	34^.^8	36^.^4	34^.^1	33^.^6	32^.^5	36^.^1	33^.^8
**3**	36^.^2	34^.^4	35^.^4	34^.^6	33^.^8	35^.^5	33^.^6	32^.^7
**4**	36^.^3	22^.^9	34^.^3	32^.^9	33^.^8	32^.^5	34^.^6	32^.^9
**5**	36^.^8	34^.^4	36^.^5	34^.^4	31^.^5	32^.^7	33^.^7	33^.^5
**6**	36^.^9	20^.^9	36^.^2	35^.^2	*Empty*	*Empty*	*Empty*	*Empty*
**7**	37^.^2	34^.^8	NEG	NEG	NEG	NEG	NEG	NEG
**8**	37^.^2	35^.^3	36^.^6	35^.^0	35^.^4	35^.^7	35^.^8	34^.^1
**9**	37^.^6	33^.^3	38^.^5	35^.^6	37^.^1	39^.^6	37^.^1	NEG
**10**	38^.^0	NEG	39^.^5	38^.^2	35^.^0	NEG	NEG	NEG
**11**	38^.^1	34^.^0	NEG/NEG^a^	37^.^4/NEG^a^	NEG	40^.^8	NEG	35.4
**12**	38^.^3	35^.^5	38^.^7	37^.^5	NEG	NEG	NEG	NEG
**13**	38^.^3	36^.^4	39^.^6	36^.^6	36^.^5	NEG/38^.^0^a^	37^.^4/35^.^8^a^	NEG/37^.^3^a^
**14**	38^.^4	33^.^0	39^.^3	34^.^5	34^.^6	39^.^8	32^.^9	37^.^3
**15**	38^.^4	33^.^4	39^.^4	37^.^5	NEG	38.7/37.8^a^	NEG/38.0^a^	NEG/NEG^a^
**16**	38^.^4	33^.^6	37^.^3	37^.^8	34.8	NEG/35.7^a^	35.3/35.9^a^	NEG/34.5^a^
**17**	38^.^6	34^.^8	NEG/38.9^a^	36^.^8/39^.^9^a^	NEG	38^.^0	NEG	35^.^7
**18**	38^.^9	34^.^2	36^.^8	36^.^9	35^.^1	37^.^7	34^.^9	34^.^1
**19**	39^.^1	35^.^5	39^.^0	36^.^1	37^.^3	NEG/36^.^8^a^	37^.^8/NEG^a^	NEG/36.5^a^
**20**	39^.^2	NEG	NEG	NEG	NEG	NEG/NEG^a^	NEG/NEG^a^	36.7/36.2^a^
**21**	39^.^2	35^.^5	38^.^2	36^.^2	36^.^6	35^.^9	36^.^7	35^.^1
**22**	39^.^4	33^.^2	37^.^7	37^.^9	36^.^3	37^.^0	36^.^3	36^.^6
**23**	39^.^5	33^.^9	39^.^6/NEG^a^	NEG/NEG^a^	NEG	NEG/NEG^a^	37.5/36.6^a^	NEG/NEG^a^
**24**	40^.^3	NEG	38^.^1	37^.^1	37^.^4	NEG/36^.^0^a^	NEG/36^.^4^a^	35^.^2/35^.^1^a^
**25**	40^.^3	33^.^9	NEG/37.7^a^	35^.^5/37^.^6^a^	NEG	34^.^1	36^.^9	NEG
**26**	40^.^4	NEG	40^.^1/38^.^0^a^	NEG/NEG^a^	NEG	26^.^9/NEG^a^	NEG/NEG^a^	NEG/NEG^a^
**27**	40^.^4	NEG	NEG	NEG	NEG	NEG	NEG	NEG
**28**	40^.^8	34^.^7	NEG/NEG^a^	37^.^9/NEG^a^	NEG	NEG	NEG	NEG
**29**	40^.^8	28^.^9	37^.^4	34^.^2	35^.^5	34^.^5/35^.^0^a^	43^.^3/36^.^4^a^	NEG/35^.^1^a^
**30**	40^.^9	34^.^2	NEG/NEG^a^	37^.^7/NEG^a^	NEG	37^.^4	40^.^2	36^.^5
**31**	41^.^0	32^.^8	NEG/38^.^7^a^	34^.^1/35^.^1^a^	37^.^1	34^.^5	34^.^2	NEG
**32**	41^.^1	34^.^4	39^.^7/38^.^7^a^	NEG/NEG^a^	NEG	35.5/NEG^a^	NEG/NEG^a^	NEG/36^.^6^a^
**33**	41^.^2	36^.^1	39^.^6	36^.^3	37^.^2	33^.^9	35^.^9	36^.^3

^a^Ct value from repeat testing with Da An and Liferiver Assays.

**Fig 1:**
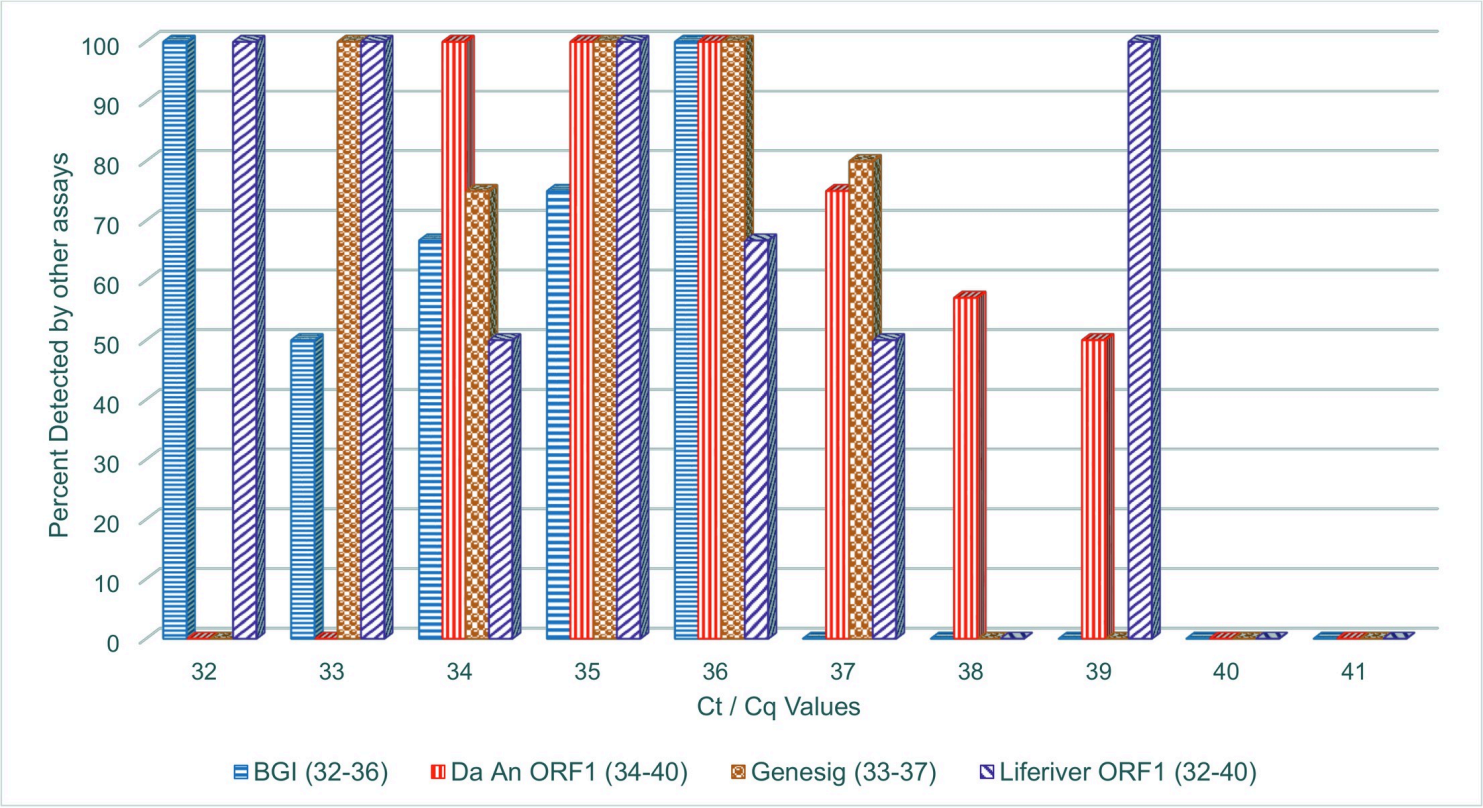
Concordant detection (%) by ALL four assays across the range of Ct/Cq values. *Note The kits gave a varied range of Ct values for the same samples. Not all kits gave Ct values across the whole range from 36–41. The Ct range for each kit is specified in the legend. Thus, no bars for some kits at particular Ct values, should not be mistaken as sample not being detected by the other kits.

From Fig 1, all samples with BGI Ct values at 32 were detected 100 percent by the other three assays, but at Ct = 33, not all samples detected by the BGI assay was detected by the other assays. The Da An Gene assay had 100% concordant detection for Ct 34 to 36, but at Ct = 37, some samples detected by the Da An Gene assay was not detected by the other assays. Similarly, Genesig assay had concordant detection till Ct = 34. Finally, the Liferiver assay had concordant detection on other assays till Ct-34 when some positive by the Liferiver assay were not detected by the other three assays.

## Discussion

All assays were sensitive to the high positive samples, correctly identifying them, but performance was varied for samples of high Ct values and probably low viral copies. Three of the assays correctly identified all the SARS-CoV-2 negative samples. However, two of the assays identified one different negative sample as positive or inconclusive. One negative sample had Ct values close to the Da An assay cut-off (39^.^9/37^.^2). This kind of error could be prevented by empirically determining an in-house cut-off, often more stringent than the manufacturer recommended cut-off. Liferiver assay picked only the N-gene and E-gene for another negative sample, without the critical ORF1ab gene detected, thus the test was inconclusive. In addition, the study sample size is small, and our estimation of sensitivity, specificity and accuracy could be impacted as shown by the wide 95% confidence intervals. Though not statistically significant, it appears the ORF1 target was detected more than the N-gene or E-gene targets (Table 3).

Given that this virus is novel now, the varied performance of the assays, especially on supposedly low-positive samples, is expected as the dynamics of the pathogenesis of SARS-CoV-2 in different population had not been fully elucidated. The Tib MolBiol assay used as the comparator has a revised cut-off of 36^.^0, where samples with Ct < 36^.^0 are considered positive and eligible to further test with the RdRP assay. This study shows that 52% of these samples with Ct > 36^.^0 and considered negative, were determined as concordant positive for SARS-CoV-2 by the other four assays. If these are genuinely positive, a lot of false negatives would have been left in the population, which may fuel the spread of the virus.

The Da An, Liferiver and Tib MolBiol assays utilise 5 μL of sample RNA, while Genesig and BGI use 8 μL and 10 μL of sample RNA, respectively. As viral copies may be low in these borderline positive samples, the higher volume of the sample taken may be instrumental in the ability of the Genesig, and BGI assays to detect more low positive samples. Thus, in-house optimization of assay RNA sample volumes could guide in its use for detecting all categories of samples. This is important given the high turnover in diagnostic assays for SARS-CoV-2 at the time of this study. Besides, the BGI assay seems well optimised as the Ct values are often much lower than their cut-off of 38^.^0. In the evaluation by FIND [5], the BGI assay Ct value of 32^.^43 for the lowest sample dilution was the least Ct recorded for that concentration by all the assays evaluated. Our study further demonstrated that samples with Ct above 33^.^0 on BGI tend to have a variable performance on the other assays. Thus, in-house optimization of the assays and where possible RNA sample volumes could guide in its use for detecting all categories of samples. This is imperative since different real-time PCR assays are in use globally.

Fig 1 shows the Ct/Cq values at which each assay first has a positive concordance issue. This might indicate at which Ct/Cq value to repeat samples positive to ensure the signal is consistent. With the global shortage of diagnostic assays and consumables, we are experiencing a high turn-over in diagnostic assays available for use. The data presented here may explain some inconsistency in test results from different assays, and why some patients may seem to resolve their infection earlier than expected. Therefore, our findings might help laboratories using these assays to decide on an empirical cut-off that may be more stringent than the cut-off recommended by the manufacturer. In addition, supervisory review of test data and curves could be reinforced as real-time PCR software may give Ct/Cq values though the graph maybe non-sigmoidal or not representative of the Ct/Cq value rendered. Depending on the assay quality assurance procedures, when the graph does not match the Ct/Cq value rendered, an experienced supervisor may ask for repeat testing to preclude releasing false positive results. Technical support from supervising agencies may help, just as the Nigeria Centre for Disease Control (NCDC) sent staff to evaluate and certify testing laboratories before they joined the testing network. The NCDC further produced and distributed standard procedures and working guides.

A lot of issues can affect PCR assay performance and thus, this study has some limitations [2]. As the differences in performance were mostly around samples with low viral copies, random sampling in picking 5–10 μl from a 50–60 μl eluted sample RNA may account for some of the differences. Still, efforts were taken in this evaluation to mitigate their impact by using the same preserved sample set over a short time interval to minimise efforts of freeze-thaws. This evaluation used a limited sample size to conserve reagents due to the global shortage of reagents and consumables. Thus, our estimates of key parameters such as sensitivity and specificity may not be accurate due to the small sample size.

The SARS-CoV-2 is novel; hence, diagnostic assays will have variable performance, especially on viral strains from different regions and more so those detecting multiple targets. One key factor to hold onto is consistency. It is imperative therefore that facilities empirically define a cut-off to accept samples as positive, and a range to repeat testing to confirm a positive signal is consistent.
